# A Metabolomics-Based Study on the Discriminative Classification Models and Toxicological Mechanism of Estazolam Fatal Intoxication

**DOI:** 10.3390/metabo13040567

**Published:** 2023-04-17

**Authors:** Xiaohui Dai, Rui Bai, Bing Xie, Jiahong Xiang, Xingang Miao, Yan Shi, Feng Yu, Bin Cong, Di Wen, Chunling Ma

**Affiliations:** 1 Hebei Key Laboratory of Forensic Medicine, Collaborative Innovation Center of Forensic Medical Molecular Identification, College of Forensic Medicine, Hebei Medical University, Shijiazhuang 050017, China; 2 Research Unit of Digestive Tract Microecosystem Pharmacology and Toxicology, Chinese Academy of Medical Sciences, Shijiazhuang 050017, China; 3 Forensic Science Centre of WATSON, Guangzhou 510440, China; 4 Shanghai Key Laboratory Medicine, Department of Forensic Toxicology, Shanghai Forensic Service Platform, Academy of Forensic Science, Shanghai 200063, China

**Keywords:** estazolam, fatal intoxication, metabolomic, LC-MS, mechanism validation

## Abstract

Fatal intoxication with sedative-hypnotic drugs is increasing yearly. However, the plasma drug concentration data for fatal intoxication involving these substances are not systematic and even overlap with the intoxication group. Therefore, developing a more precise and trustworthy approach to determining the cause of death is necessary. This study analyzed mice plasma and brainstem samples using the liquid chromatography-high resolution tandem mass spectrometry (LC-HR MS/MS)-based metabolomics method to create discriminative classification models for estazolam fatal intoxication (EFI). The most perturbed metabolic pathway between the EFI and EIND (estazolam intoxication non-death) was examined, Both EIND and EFI groups were administered 500 mg of estazolam per 100 g of body weight. Mice that did not die beyond 8 hours were treated with cervical dislocation and were classified into the EIND groups; the lysine degradation pathway was verified by qPCR (Quantitative Polymerase Chain Reaction), metabolite quantitative and TEM (transmission electron microscopy) analysis. Non-targeted metabolomics analysis with EFI were the experimental group and four hypoxia-related non-drug-related deaths (NDRDs) were the control group. Mass spectrometry data were analyzed with Compound Discoverer (CD) 3.1 software and multivariate statistical analyses were performed using the online software MetaboAnalyst 5.0. After a series of analyses, the results showed the discriminative classification model in plasma was composed of three endogenous metabolites: phenylacetylglycine, creatine and indole-3-lactic acid, and in the brainstem was composed of palmitic acid, creatine, and indole-3-lactic acid. The specificity validation results showed that both classification models distinguished between the other four sedatives–hypnotics, with an area under ROC curve (AUC) of 0.991, and the classification models had an extremely high specificity. When comparing different doses of estazolam, the AUC value of each group was larger than 0.80, and the sensitivity was also high. Moreover, the stability results showed that the AUC value was equal to or very close to 1 in plasma samples stored at 4 °C for 0, 1, 5, 10 and 15 days; the predictive power of the classification model was stable within 15 days. The results of lysine degradation pathway validation revealed that the EFI group had the highest lysine and saccharopine concentrations (mean (ng/mg) = 1.089 and 1.2526, respectively) when compared to the EIND and control group, while the relative expression of SDH (saccharopine dehydrogenase) showed significantly lower in the EFI group (mean = 1.206). Both of these results were statistically significant. Furthermore, TEM analysis showed that the EFI group had the more severely damaged mitochondria. This work gives fresh insights into the toxicological processes of estazolam and a new method for identifying EFI-related causes of mortality.

## 1. Introduction

Sedative-hypnotic drug intoxication occurs commonly with increasing death rates nowadays in forensic practice. According to AAPCC (American Association of Poison Control Centers) annual reports for the past five years, sedatives/hypnotics/antipsychotics cause the highest number of deaths of all drug intoxication deaths, and the exposure rate of these drugs is rising [1,2,3,4,5]. Similar literature has been reported in Europe and Japan [6,7]. Deaths from veterinary sedative-hypnotic drugs have also been reported [8,9].

However, forensic pathology practice has shown that the autopsy findings of many fatal intoxication deaths caused with prescription and illicit drugs, such as pulmonary edema and bladder distension urine, are not specific and are referred to as negative autopsies [10] because the results do not contribute to identifying the unknown death cause. Moreover, in the few studies focusing on lethal plasma concentrations of sedative-hypnotic drugs, for multiple sedative-hypnotic drugs, there is an overlap of plasma concentrations between the fatal intoxication and reference groups or even intoxicated groups. The amount of plasma concentration data for fatal intoxication caused with some single sedative-hypnotic drugs is grossly inadequate, and lethal plasma concentrations lack systematicity and typicality [11,12]. In some studies, plasma concentrations only reflected the intake of such drugs before death [13]. In addition, postmortem drug redistribution exhibits an effect [14]. In the 5–10% of cases where reliable results cannot be achieved using conventional methods [15], developing more precise and reliable procedures for identifying the cause of death in sedative-hypnotic drug overdoses is crucial.

Metabolomics, focusing on small molecule compounds, has been widely used in forensic toxicology [15,16]. The so-called advanced molecular autopsy is the application of multi-omics approaches such as metabolomics, thus delivering the death-cause identification process to the molecular biology level [17]. The concept of thanatometabolomics has been proposed and implemented for the identification of unknown death causes, postmortem interval (PMI) estimation and screening of potential toxicological biomarkers. This process is also known as metabolomic autopsy [15]. As an alternative to standard forensic toxicology methods, the metabolomics approach can allow the confirmation of drug consumption or manipulation attempts. It also has a wide range of applications in the identification of drug and new psychoactive substance (NPS) abuse, as well as alcohol abuse. For example, the metabolites of ethyl alcohol (ethanol), which are ethyl sulfate (EthS), ethylglucuronide (EthG), phosphatidylethanol (PEth), etc., have a more extensive window of detection and can be used as suitable biomarkers for metabolomic identification [16]. In addition, some successful applications have been made in forensic analysis to identify the complex cause of death with the construction of a metabolomic classification model [18,19]. Our previous study on fatal intoxication with antipsychotic drugs also successfully achieved cause of death identification through the use of discriminative classification models [20]. Therefore, this study proposes adopting metabolomics to identify fatal intoxication caused with sedative-hypnotic drugs.

The principal experimental drug in this study was estazolam, a traditional benzodiazepine sedative-hypnotic drug that produces anticonvulsant, anxiolytic and sedative-hypnotic effects mainly through gamma-amino-butyric acid (GABA) receptor modulation in the central nervous system [21], and it is one of the top-ranked potentially inappropriate medications [22], and is commonly used for drug-related suicide. However, estazolam is like other sedative and hypnotic drugs; there is also a lack of data from systematic studies [2].

In this study, we propose to use a metabolomic approach to screen for endogenous potential biomarkers of fatal intoxication with estazolam and to construct two classification models in the plasma and brainstem tissue samples, respectively, to address the issue that the existing lethal plasma concentration data cannot accurately and effectively explain the cause of death or provide objective molecular evidence for the identification of the cause of death of fatal intoxication with this drug in forensic practice. Meanwhile, the most perturbed metabolic pathways in brainstem tissues of estazolam fatal intoxication mice were screened to explore the drug’s new toxicological mechanism further.

## 2. Materials and Methods

### 2.1. Chemicals and Reagents

Estazolam and zaleplon were purchased from Shanxi Xinbaoyuan Pharmaceutical Co., Ltd. (Datong, China). Diazepam and nitrazepam were purchased from Jiangsu Nhwa Pharmaceutical Co., Ltd. (Xuzhou, China). Sodium pentobarbital was purchased from Sigma Chem. Co., (St. Louis, MO, USA). Saccharopine (CAS Number: 997-68-2) standards were purchased from Shanghai yuanye Bio-Technology Co., Ltd (Shanghai, China). Lysine (CAS Number: 56-87-1), Proadifen Hydrochloride (SKF525A) (CAS Number: 62-68-0) standard were purchased from CSNpharm Inc. (Chicago, IL, USA), estazolam (CAS Number: 29975-16-4) was purchased from National Institutes for Food and Drug Control of China (Beijing, China). Methanol, acetonitrile, and formic acid (HPLC grade) were purchased from Dikma Technology Inc. (Foothill Ranch, CA, USA). The Milli-Q system (Millipore, Burlington, MA, USA) was used to purify the deionized water.

### 2.2. Animals Diets and Grouping

All experimental animal protocols of this study were approved by the institutional ethics committee of Hebei Medical University (No. 20180071, 2018-04-15). All CD1(ICR) mice (7–8 weeks, female: 32–37 g, male: 38–42 g) were purchased from Liaoning Changsheng Biotechnology Co., Ltd. (Shenyang, China), and kept under a standard 12:12 h light/dark cycle environment with 23 ± 2 °C and 55 ± 5% relative humidity. All animals were fed with specific pathogen-free-grade chow for a week and fasted overnight (with libitum access to water) before experimentation. Briefly, 180 mice in 23 groups were involved in this study.

To achieve an appropriate intragastric administration volume of 0.1–0.2 mL per 10 g of body weight, drug concentrations were adjusted before use. According to the above experimental design, quantitative plasma concentration analysis was first performed in the estazolam fatal intoxication (EFI) and estazolam intoxication non-death (EIND). Combining previous studies and our protocol, these two groups were administered at a concentration of 500 mg/100 g (6 × LD50) [23]. The EIND group mice (*n* = 10, Female: Male = 1:1) were poisoned, survived for more than 8 h and treated with cervical dislocation, but the EFI group mice (*n* = 10, F:M = 1:1) died within 8 h of administration. The blood remaining after plasma concentration quantification was used for subsequent metabolomics experiments.

In the metabolomic analysis session, EFI (*n* = 10, F:M = 1:1) was the experimental group, and four NDRD (non-drug related death, *n* = 10, respectively, F: M = 1:1) mice models were brought in as controls, simulating the death cases associated with hypoxia in forensic practice, including cervical dislocation (CD), drowning (DR) [24], mechanical asphyxia (MA) [25] and acute hemorrhagic shock (HS) [26]. The DR, MA and HS groups were given cervical dislocation to relieve pain at the time of almost dying in all three groups. A new batch of data matching the first five categories was presented to verify the prediction ability of the classification model. Moreover, four other sedative-hypnotic drug fatal intoxication models consistent with EFI, and both simulated cases of extreme drug fatal intoxication of iatrogenic toxicant in forensic practice (*n* = 10, respectively, F:M = 1:1) were: diazepam (280 mg/100 g, 5 × LD50 [27]), nitrazepam (275 mg/100 g, 5 × LD50 [28]) and zaleplon (280 mg/100 g: refer to the dosage of diazepam), and sodium pentobarbital (20 mg/100 g, ≈1.6 × LD50 [29]). Then, to assess the classification model’s sensitivity, three other estazolam dose groups (*n* = 10, respectively, F:M = 1:1) were introduced, namely 3 × LD50 (250 mg/100 g), 50 × therapeutic (2.59 mg/100 g) and 100 × therapeutic (5.17 mg/100 g) of estazolam [30] groups, of which the latter two groups were administered for 24 h, then euthanized by cervical dislocation. Then, the EFI (*n* = 8, F: M = 1:1) group and three control groups (CD, DR, MA, *n* = 6, respectively, F:M = 1:1) were introduced, and their plasma was stored at 4 °C for 0, 1, 5, 10, 15 and 20 days to assess the classification model’s stability. All the above metabolomics groups used plasma and brainstem tissues for differential metabolite screening and classification model construction and evaluation, except for the stability experiments where only plasma was used, and their brainstem samples were used for subsequent molecular experiments.

Finally, brainstems from the EFI and EIND groups (*n* = 10, respectively, F:M = 1:1) were used for metabolic pathway enrichment analysis, and lysine and saccharopine content in the brainstem were measured together with the CD group (*n* = 10, F:M = 1:1), and a qPCR quantification of the key enzymes of the metabolic pathway was done, as well as a morphological electron microscopic analysis. The objective was to investigate new estazolam toxicity pathways.

The experimental grouping information is listed in Supplementary Materials Table S1.

### 2.3. Blood and Brainstem Tissues Sample Collection

For all animals used for metabolomics analysis, abdominal aortic blood was collected immediately following cervical dislocation or death and transferred into heparin-treated 1.5 mL centrifuge tubes on ice; blood samples were centrifuged at 8000× *g* for 10 min at 4 °C to obtain supernatant plasma [31]. In the EFI and EIND groups, after blood was drawn from the abdominal aorta, 200 µL of whole blood was administered initially for plasma concentration quantification. All plasma samples were snap-frozen in liquid nitrogen and stored at −80 °C until use. For stability evaluation, 8 EFI (F:M = 1:1) and 18 NDRD plasma samples (drowning: cervical dislocation: mechanical asphyxia = 1:1, F:M = 1:1) were stored at 4 °C from the 1st to 20th days. About 60 μL plasma was absorbed in the 0, 1st, 5th, 10th, 15th, and 20th days respectively. All plasma samples obtained this way were restored at −80 °C before pretreatment.

Brainstem tissue was quickly obtained from each animal group after blood sampling and was snap-frozen with liquid nitrogen and crushed, weighed into 50 mg portions at low temperature, and placed in 2 mL frozen storage tubes at −80 °C until use. Each brainstem sample was divided into 2–3 copies, and each was 50 mg.

### 2.4. Blood and Brainstem Tissue Metabolite Extraction

Pretreatment of plasma and brainstem tissue samples for non-targeted metabolomics analysis was based on and modified from the methods in the literature [31,32]. Briefly, 100 μL of the plasma samples were added to the tubes with 300 μL ice-cold methanol (Vsample: Vextraction = 1:3) after thawing at 4 °C. Each sample was then vortexed for 30 s, sonicated for 10 min in an ice-water bath, and incubated for 20 min at −20 °C for protein precipitation. The mixing solution was centrifuged at 12,000× *g* for 10 min at 4 °C. The resulting supernatants were transferred to LC-MS vials for a LC-HR MS/MS analysis. Furthermore, the quality control (QC) sample was mixed with 10 µL supernatant taken from each sample.

Pretreatment of brainstem tissue samples was as follows. Briefly, 50 mg of brainstem tissue was thawed at 4 °C, 300 μL of ice-cold methanol was added, and an appropriate amount of grinding beads were added. This was ground at −4 °C for 90 s to form a homogenized slurry. Then, the sample was vortexed for 30 s, sonicated for 10 min in an ice-water bath, and incubated for 20 min at −20 °C to allow protein precipitation. The mixtures were centrifuged at 12,000× *g* for 10 min at 4 °C. The resulting supernatants were transferred to LC-MS vials for UPLC-MS/MS analysis. QC samples were processed as before.

### 2.5. Data Acquisition with Full Scan-MS/MS Using LC-HR MS/MS

LC-HR MS/MS analysis was performed by the Ultimate 3000 rapid separation UHPLC coupled with Q Exactive Orbitrap MS (Thermo Fisher Scientific, Waltham, MA, USA). This specific method was modified based on the scheme of Huang et al. [33]. A UPLC HSS T3 column (2.1 × 100 mm, 1.8 μm; Waters Corp., Milford, MA, USA) was used for separation. Mobile phase A was 0.1% formic acid in Milli-Q water, and mobile phase B was pure acetonitrile. The elution gradient was set as follows: 0 min, 98% A; 1 min, 98% A; 12 min, 2% A; 16 min, 2% A; 16.1 min, 98% A; 20 min, 98% A. The flow rate was 0.3 mL/min, and the injection volume was 5 μL. The total run time was 20 min. During LC/MS studies, a Q Exactive mass spectrometer was used for acquiring an MS/MS spectra on an information-dependent basis (IDA) and the full scan mode (resolution of full MS 35,000, MS2 17,500). In this mode, Xcalibur™ software (version 4.0.27, Thermo Fisher Scientific) continuously assessed MS data collected during the full scan survey and triggered the acquisition of the MS/MS spectra that exceeded the preselected criteria. The MS was equipped with a heat electrospray ionization (HESI, Thermo Fisher Scientific) source and operated under the positive/negative ionization switching mode. The ESI source conditions were set as follows: the spray voltage was +3.2 kV or −3.1 kV, Scan range 80–1200 *m*/*z*, AGC targetvalue = 1 × 10^5^, sheath gas flow rate = 35 arb, aux gas flow rate = 15 arb, and capillary temperature = 350 °C. The collision energy was 25/35/45 eV in the normalized collision energy model.

Determination of the plasma concentration, measurement of saccharopine and lysine in the brainstem tissue, quantitative real-time PCR (QuantStudio 7 Flex, Applied Biosystems, Waltham, MA, USA), and TEM (HT7800, Hitachi, Tokyo, Japan) analyses are in the Materials and Methods section of the supplementary materials.

### 2.6. Data Processing and Discriminating Component Analysis

Data preprocessing significantly impacts untargeted metabolomics analysis [34]. In this study, MS raw data files were preprocessed using CD software (version 3.1, Thermo Fisher Scientific) while applying the existing metabolomics process named “Untargeted Metabolomics with statistics detect unknowns with ID using Online Database and mzLogic”. The default parameters were the Retention Time alignment, Fill Gaps, and Peak Area Correction based on QC samples. Minimum peak intensity was changed to 5 × 10^5^ to match various compounds. The preprocessing result is a data matrix table containing retention time, exact molecular weight, annotation information and other items. The analysis included only data with similar MS2 structural features as determined with the mzCloud database to improve the metabolomics analysis accuracy.

The online software MetaboAnalyst (https://www.metaboanalyst.ca/, accessed on 15 April 2023), version 5.0, McGill University’s Xia-lab, Montreal, QC, Canada) was used for multivariate statistical analysis after pretreatment. The normalization before each data analysis was referenced to autoscaling and log transformation (base 10). PCA (principal component analysis) was done to screen the different metabolites between EFI and NDRDs in plasma and brainstem tissue samples. R language was utilized for correlation analysis of QC samples to evaluate the reproducibility and system stability of the approach. PLS-DA (partial least squares discriminant analysis) was carried out to achieve a better separation effect between groups, and the model was evaluated with 10-fold cross-validation and 100 permutation test. Then, biomarker meta-analysis was used to determine the intersection of differential metabolites between EFI group and each NDRD group [35]. The candidate differential metabolites of the classification model were screened by *p* value and Combined LogFC (fold change) value combined with mzCloud database MS2 spectrometry match score in the result matrix and Combined VIP (variable importance in projection) value obtained with PLS-DA analysis.Next, the use of biomarker analysis along with multivariate receiver operating characteristic (ROC) curve-based test analysis which utilized the linear support vector machine (SVM) algorithm was used to construct a better classification model for EFI. considering We have meanwhile taken the results of conventional univariate ROC curve analysis into consideration as well. The discriminative power and reliability of the classification models were further evaluated, and stability was verified in plasma samples. Precision (positive predictive value (PPV)), negative predictive value (NPV), Recall and F1-score (plotted with GraphPad 8, San Diego, CA, USA) were used in combination with AUC values for a comprehensive evaluation [36,37]. The cutoff value (0.8) was set with reference to Yu et al. [38].

To study the new toxicological mechanism of estazolam, the brainstem as the target organ was taken as the research object; brain tissue is the common examination material in forensic practice [16]. The student’s *t*-test and PLS-DA analysis drew the volcanic plot, and the differential metabolites between EFI and EIND groups were obtained. Then the quantitative enrichment analysis of metabolic pathways was conducted based on the differential metabolites. According to the enrichment ratio and *p* value, the lysine degradation pathway with the largest disruption was identified among all the differential pathways. LC-MS/MS analysis was used to determine the content of key metabolites in the brainstem of the lysine degradation pathway in control, EFI and EIND groups with *t*-test; qPCR was used to determine the relative expression of key enzymes in this pathway with one-way ANOVA (*p* < 0.05, GraphPad 8). To visually illustrate the new toxicological mechanism of estazolam, morphological changes in EFI and EIND brainstem tissues were compared with transmission electron microscopy (TEM) data.

## 3. Results

**Figure 1:**
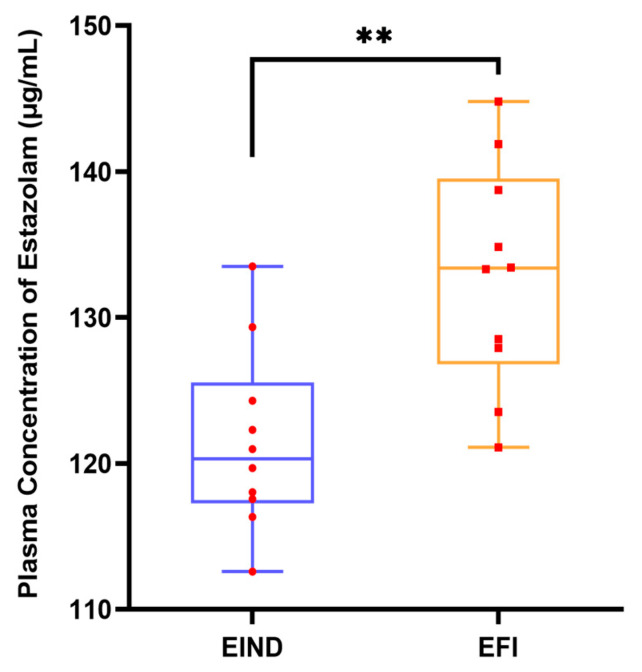
Plasma concentration of estazolam in mice from EFI and EIND groups at 6 × LD50 dose (500 mg/100 g). Notes: Quantitative results of plasma concentration of estazolam, n_animal_ = 10, respectively, female: male = 1:1, **, *p* < 0.01, are compared between EFI and EIND groups. Statistical significance was determined with the student’s *t*-test. Box plots are expressed as medians (horizontal lines in boxes), 25 to 75 percentile (boxes), and extent of data (whiskers), with red circles and red squares representing specific data points. Abbreviations: EFI: Estazolam fatal intoxication, EIND: Estazolam intoxication not-dead, and EFI: Estazolam acute intoxication death. EIND meant acute intoxication over 8 h, followed by cervical dislocation. The dose and duration of administration were the same for both groups.

There was a statistically significant distinction between the plasma concentrations of estazolam in the EFI and EIND groups, and the results were statistically significant. However, the two groups have a substantial overlap in estazolam concentrations. The outcomes are depicted in Figure 1.

### 3.1. Metabolomics Profiling of Plasma Samples in the EFI and NDRDs Mice

**Figure 2:**
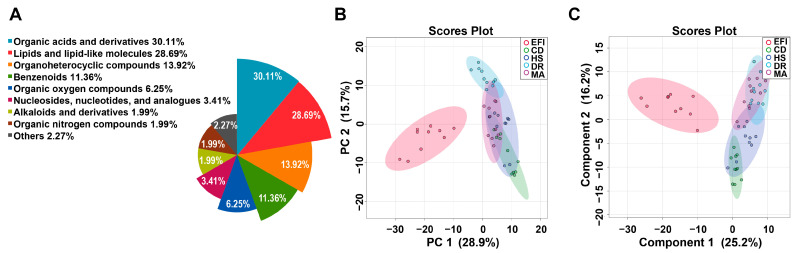
Overviews of the metabolic profiles of plasma samples in the EFI and NDRDs mice. NDRDs, including CD, DR, MA, and HS, are four animal models. n_animal_ = 10, respectively. Total 50 samples in 5 groups. (**A**) Nightingale Rose Chart of metabolite classification. (**B**) Overview of metabolic profiles of the EFI and NDRDs groups using principal component analysis (PCA) score plot. (**C**) The partial least squares discriminant analysis (PLS-DA) score plot. Abbreviations: NDRD: non-drug related death, CD: cervical dislocation, DR: drowning, MA: mechanical asphyxia, HS: acute hemorrhagic shock.

Untargeted metabolomic analysis in 50 plasma samples from the EFI group, and the four NDRDs groups (CD, DR, HS, and MA) used as negative controls, detected 392 metabolites (molecular characteristics were shown in the Excel spreadsheet of supplementary materials), of which 352 were annotated by the Human Metabolome Database (HMDB) and 40 metabolites remain unclassified at present by the HMDB. The annotated metabolites could be classified into nine chemical classes, most of which were organic acids and their derivatives (30.11%), followed by lipids and lipid-like molecules (28.69%), organic heterocyclic compounds (13.92%), benzenes (11.36%). The details are in Figure 2A. The specific data are shown in Table S2 of the Supplementary Materials.

The overview of global metabolic profiles according to the quantitative results for the metabolites in the mice plasma samples, as revealed by PCA scores plots (Figure 2B), showed obvious dissimilarities between the EFI group and the four control groups. This differentiation could be described with the first PC1, which accounted for 28.9% of the variance. The second PC (PC2) accounted for 15.7% of the variance. Figure S1A demonstrates that the correlation between QC samples is extraordinarily high and that the analytical method has excellent reproducibility and systematic stability.

Further PLS-DA analysis was performed to show better separation trends between the different groups. The PLS-DA scores plot (Figure 2C) demonstrated a good separation trend between EFI and control groups, and the four control groups were further separated. The cross-validation method with a 10-fold CV algorithm was applied to calculate the Q2 to evaluate the PLS-DA model. Figure S1B shows that the model had an R2 of up to 0.98675 and a Q2-value of 0.89801 for the five components, indicating its high predictive power [35]. The results of the 100 permutation test (*p* < 0.01, Figure S1C) showed that the model was fitting well, and it had high stability.

### 3.2. Classification Model Screening and Verification in EFI Plasma Samples Relative to NDRDs Mice

**Figure 3:**
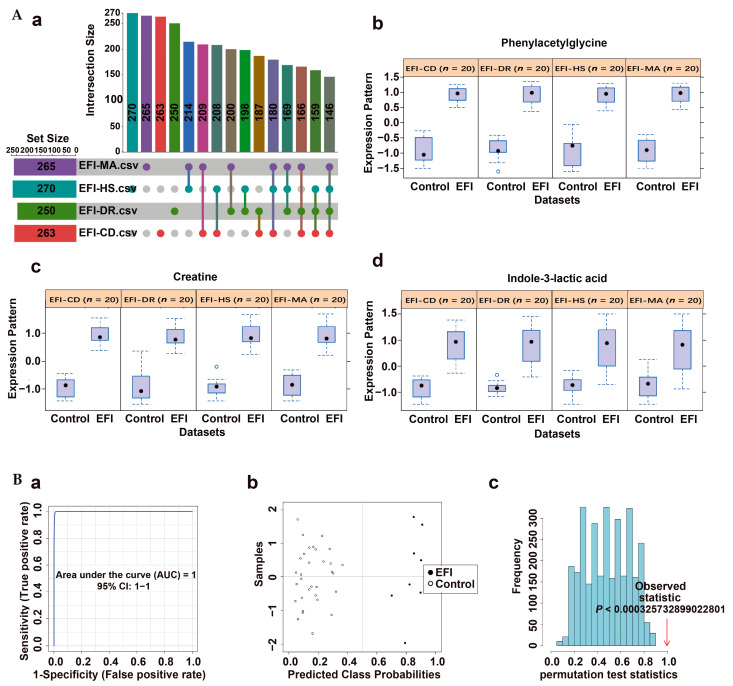
Screening classification model in plasma samples from the EFI group relative to the NDRDs group. (**A**) (**a**) Upset plots obtained with biomarkers meta-analysis of EFI and four NDRD groups (total of 5 groups, n_animal_ = 10, respectively), with 146 common differential metabolites. (**b**–**d**) The classification model consisted of three candidate differentials, phenylacetylglycine, creatine and indole-3-lactic acid, and they were significantly up-regulated in the EFI group. (**B**) Discriminatory ability evaluation of the EFI classification model in the training set (n_EFI_ = 8, n_NDRDs_ = 32). (**a**) ROC plot: AUC = 1 (95% CI: 1−1) (**b**) Confusion matrix plot showing samples without misclassification. (**c**) Classification model with 100 permutation-test plots, *p* < 0.01. Notes: (**B**) construction and evaluation of classification models using multivariate ROC curve analysis based on linear SVM algorithm. (**a**–**c**) in (**B**) respectively represent: (**a**) the area under the ROC (AUROC (AUC)) of the classification model in different datasets contains the AUC-value and its 95% confidence interval (CI). (**b**) The confusion matrix showed the average of predicted class probabilities between EFI and different controls; the classification boundary was at the center dotted line of x = 0.5. (**c**) Permutation tests using the model’s predictive accuracy as a measure of performance. The plots showed the actual observed AUC of all permutations and *p* value.

The common differential metabolites between the EFI group and the four NDRDs groups were screened based on the results of PLS-DA analysis using biomarker meta-analysis using the online software MetaboAnalyst 5.0, and 146 differential metabolites were obtained as shown in the upset plot (Figure 3A(a)). Fourteen candidate differential metabolites were obtained after combining the compound annotation information of the mzCloud database MS2 spectrometry match score (>85), *p* value (<0.05), combined LogFC (>1, <−1) and combined VIP-value (>1) obtained from PLS-DA analysis for comprehensive evaluation (Table 1). A multivariate ROC curve based on a linear SVM algorithm in the biomarker analysis was applied to build the EFI classification model. The screening process was described in Results 2.1 and Table S3 of the Supplementary Materials. Moreover, a high discriminability classification model consisting of three candidate differential metabolites with high predictive power, phenylacetylglycine, creatine and indole-3-lactic acid, was obtained with iterative validation, combined with the classical univariate ROC curve analysis results (AUC, 95% CI). The *p* values are shown in Table 2. Phenylacetylglycine, creatine and indole-3-lactic acid were significantly up-regulated in the EFI, compared with the four NDRDs groups (Figure 3A(b–d)). The specific procedure used 80% of the above 50 samples as the training set [39] (n_EFI_ = 8, n_NDRDs_ = 32). The results showed that the classification model consisting of these three candidate differentials based on 100 cross-validations had an AUC value equal to 1 (Figure 3B(a)). The model had a strong discriminatory ability. The confusion matrix plot (Figure 3B(b)) distribution also showed this. Then, the model was evaluated with a permutation test (*p* < 0.01), and the results were statistically significant (Figure 3B(c)), demonstrating the classification model’s stability and robustness. The validation set (n_EFI_ = 2, n_NDRDs_ = 8), consisting of the remaining 20% of samples as new samples, showed that all samples were correctly classified, as shown in Table S4.

To verify the discriminative ability of the classification model, a new set of samples consistent with the above modeling process was introduced as a test set still evaluated with a multivariate ROC curve based on a linear SVM algorithm. The AUC (=1) and the confusion matrix plot showed that the classification model still had a high discriminative ability, and the result of the 100-permutation test (*p* < 0.01) showed that the model had good predictive power (Figure S1D(a–c)).

**Figure 4:**
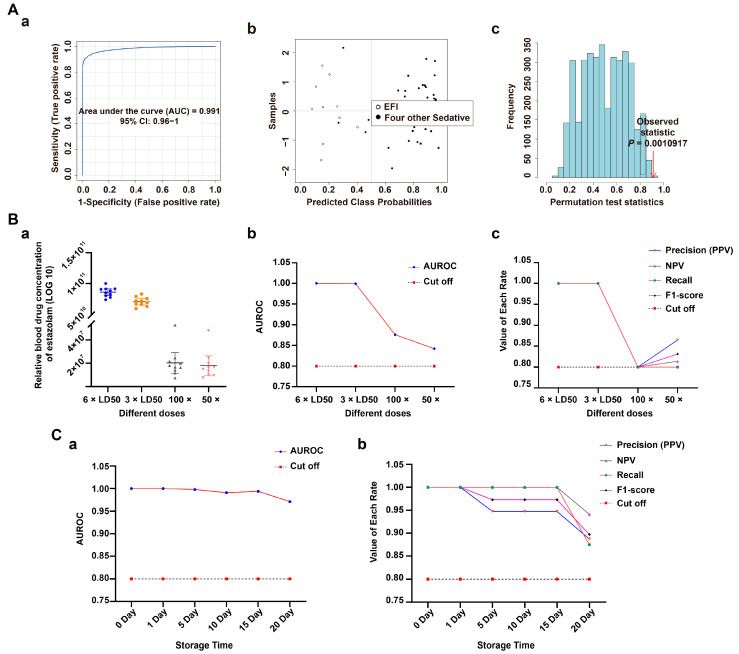
Validation of classification model in plasma samples. (**A**) Specificity evaluation of the EFI classification model relative to four other sedative-hypnotic drugs’ fatal intoxication models (zaleplon, diazepam, nitrazepam and sodium pentobarbital, n_animal_ = 10, respectively): (**a**) ROC plot: AUC = 0.991 (95% CI: 0.96−1); (**b**) confusion matrix plot showing samples with few misclassifications; (**c**) classification model with 100 permutation-test plots, *p* < 0.01. (**B**) Sensitivity evaluation of the EFI classification model relative to other different dose groups of estazolam (3 × LD50, 100 × and 50 × therapeutic dose groups, n_animal_ = 10, respectively.): (**a**) Relative plasma concentration of estazolam (LOG10) in different dose groups. The blue circles, orange squares, gray and pink triangles in the plot represent the relative blood drug concentrations of estazolam in their groups, respectively. (**b**) line plot of AUC-value, cutoff = 0.8; (**c**) line plot of the Precision (PPV), NPV, Recall and F1-score, cutoff = 0.8. (**C**) Classification model stability evaluation over time (0, 1, 5, 10, 15, and 20 days) in EFI (n_animal_ = 8) and NDRDs (CD, DR, MA. n_animal_ = 6, respectively). Line plot of AUR-value: (**a**) Precision (PPV), NPV, Recall and F1-score value (**b**) over time, cut-off = 0.8. Notes: Precision (PPV) = tp/(tp + fp); NPV = tn/(tn + fn); Recall = tp/(tp + fn); F1-score = 2 × PRE × REC/(PRE + REC); tp: total number of true positive samples; tn: total number of true negative samples, fp: Total number of false positive samples, fn: Total number of false negative samples. Because the number of EFI groups differed significantly from each control group, the above rates were calculated by weighting. Abbreviations: PPV: positive predictive value, NPV: negative predictive value, precision: PRE, recall: REC.

In this study, the discriminability and reliability of the classification model were further evaluated at three levels: specificity, sensitivity, and stability. The specificity of the classification model was first assessed by introducing four other sedative-hypnotic drug lethal intoxication models (zaleplon, diazepam, nitrazepam, and sodium pentobarbital) as a control group for EFI. The ROC test based on the SVM algorithm was used, and the results are shown in Figure 4A(a–c): AUC = 0.991, the confusion matrix plot had almost no misclassified samples, and *p* < 0.01 for the 100-permutation test. The classification model has a strong specificity and high predictive power. In addition, it showed high specificity in differentiating from the other four models of toxicant fatal intoxication (Figure S1E(a–c)).

Second, to further verify the sensitivity of this classification model, three other dosing groups (3 × LD50, 100 × and the 50 × therapeutic dose intoxication group) were introduced for the above ROC testing. The relative plasma concentrations of the four groups of estazolam are shown in Figure 4B(a), specific data were shown in Table S5, and the changes in AUC values are in Figure 4B(b). The 100 × and 50 × therapeutic dose intoxication groups were also well differentiated from the four NDRD groups relative to the extreme dose model (6 × LD50, 3 × LD50) with AUC-values of 0.876 and 0.842, respectively, which are both higher than 0.8. While the line plots of Precision (PPV), NPV, Recall and F1-score of the four groups were consistent with the trend of the AUC results, the above four rates were greater than or equal to 0.8 for both the 100 × and 50 × groups. The Precision (0.8649), NPV (0.814) and F1-score (0.8312) for the 50 × group were slightly higher than those for the 100 × group (Figure 4B(c)); related data are shown in Table S6 (AUC-value, Precision (PPV), NPV, Recall, and F1-score). Thus, the discriminatory ability of the classification model is also very high in the low-dose group. The classification model shows high sensitivity.

Finally, stability validation determines whether the above classification model can be used as a prerequisite for forensic diagnosis. In this study, four sets of samples (n_EFI_ = 8, n_CD, DR, MA_ = 6, respectively) were stored at 4 °C for 0, 1, 5, 10, 15 or 20 days. We deleted HS because it had a very similar metabolic profile to MA (Figure 2B). As shown in the line graphs of the changes in AUC on days 0, 1, 5, 10, 15, and 20, the discrimination ability of the classification model showed a slow decline over time, but the AUC value on day 20 was still high at 0.971—very close to 1 (Figure 4C(a)). This trend was also reflected in the line graph of the temporal change of the Precision (PPV), NPV, Recall and F1-score of the model in these 20 days, where only the sixth time point (day 20) had a Precision (PPV) = 0.8873, Recall = 0.875 and F1-score = 0.8811 slightly lower than 0.9, and the NPV = 0.9412 at this point, but these numbers were also much higher than 0.8 (Figure 4C(b)). Related data are shown in Table S7. The model was very stable.

### 3.3. Metabolomics Profiling of Brainstem Tissue Samples in the EFI and NDRDs Mice

**Figure 5:**
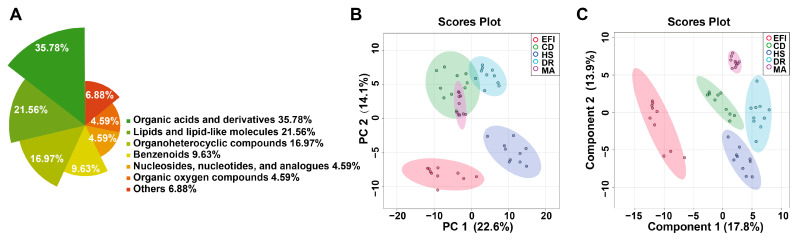
Overviews of metabolic profiles of brainstem tissue samples in the EFI and NDRDs mice. There were fifty mice in groups, n_animal_ = 10, respectively. (**A**) Nightingale Rose Chart of metabolite classification. (**B**) PCA score plot. (**C**) PLS-DA score plot.

Respiratory depression is one of the important toxicological mechanisms of estazolam lethality; therefore, additional non-targeted metabolomic analysis was performed on the target organ brainstem in the same groups as the plasma samples, and 244 metabolites of were detected, 218 of which were annotated by the Human Metabolome Database (HMDB), while 26 metabolites remained unclassified. Molecular characteristics are shown in the Excel spreadsheet of the supplementary materials. The annotated metabolites can be grouped into seven chemical classes with a similar percentage profile to the plasma results; the details are illustrated in Figure 5A. Specific data were shown in Table S2.

The overview of global metabolic profiles according to the quantitative results for the metabolites in the mouse brainstem tissue samples, as revealed with PCA score plots (Figure 5B), showed obvious dissimilarities between the EFI and the four control groups. The result is similar to the situation in the plasma sample group. This differentiation could be described with the first PC1, which accounted for 22.6% of the variance. The second PC (PC2) accounted for 14.1% of the variance. Figure S2A showed that the correlation between QC samples was extremely high, and the analytical method had good reproducibility and system stability.

PLS-DA analysis was further performed to show better separation trends between the different groups, and the results were essentially the same as for the plasma samples. However, the four control groups showed better separation trends (Figure 5C). The results of cross-validation based on 10-fold CV showed that the model had a high R2 of 0.98959 and a Q2-value of 0.85946 for the five components, which had good predictive power (Figure S2B). The 100 times permutation test result (*p* < 0.01) showed that the model had high predictive power and good stability (Figure S2C).

### 3.4. Classification Model Screening and Verification in EFI Brainstem Tissue Samples Relative to NDRDs Mice

**Figure 6:**
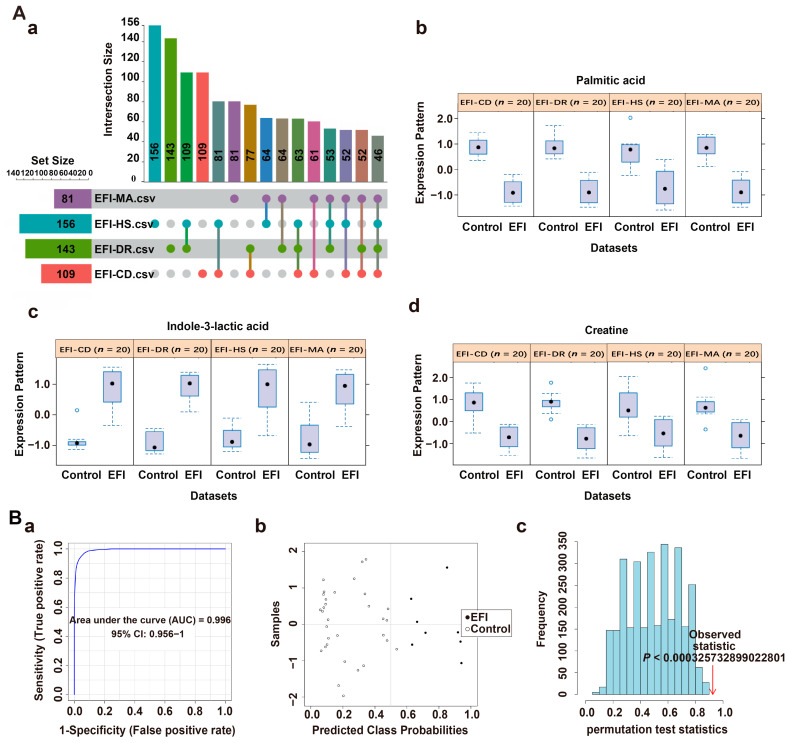
Screening of classification model in brainstem tissue samples from the EFI group relative to the NDRDs group. (**A**) (**a**) Upset plots obtained with biomarker meta-analysis of EFI and four NDRD groups (total of 5 groups, n_animal_ = 10, respectively), with 46 common differential metabolites. (**b**–**d**) The classification model consisted of three candidate differentials: palmitic acid, creatine, and indole-3-lactic acid; indole-3-lactic was up-regulated in the EFI group, and palmitic acid and creatine were down-regulated. (**B**) Discriminatory ability evaluation of the EFI classification model relative to the NDRDs groups: (**a**) ROC plot: AUC = 0.996 (95% CI: 0.956−1); (**b**) the confusion matrix plot showed that one case was wrongly diagnosed as EFI. (**c**) 100 permutation-test plot, *p* < 0.01.

Consistent with plasma sample data processing, 46 common differential metabolites (Figure 6A(a)) and six candidate differential metabolites were screened from the EFI (*n* = 10) relative to the four NDRDs (*n* = 10, respectively) groups. (Table 3). Furthermore, a classification model consisting of palmitic acid, creatine, and indole-3-lactic acid was developed in brainstem tissue samples. The screening process was described in Results 2.1 and Table S8 in the Supplementary Materials. The indole-3-lactic acid was significantly up-regulated in the EFI group, while palmitic acid and creatine were down-regulated (Figure 6A(b–d)). The AUC-values, 95% CI and *p*-values of the three differential metabolites found through classical univariate ROC curve analysis are shown in Table 4. The specific procedure was to use 80% of the above 50 samples as the training set (n_EFI_ = 8, n_NDRDs_ = 32), and the results showed that the AUC value was equal to 0.996 (Figure 6B(a)), which shows the model had a very strong discriminative power. The confusion matrix plot (Figure 6B(b)) also showed that the model had strong predictive power, the permutation-test (*p* < 0.01) result was statistically significant (Figure 6B(c)), and the classification model had strong stability and robustness. The remaining 20% of samples were used as the validation set, and the result showed that all samples were correctly classified (Table S9). The validation session of the test set was dispensed based on the experience of plasma sample data processing.

**Figure 7:**
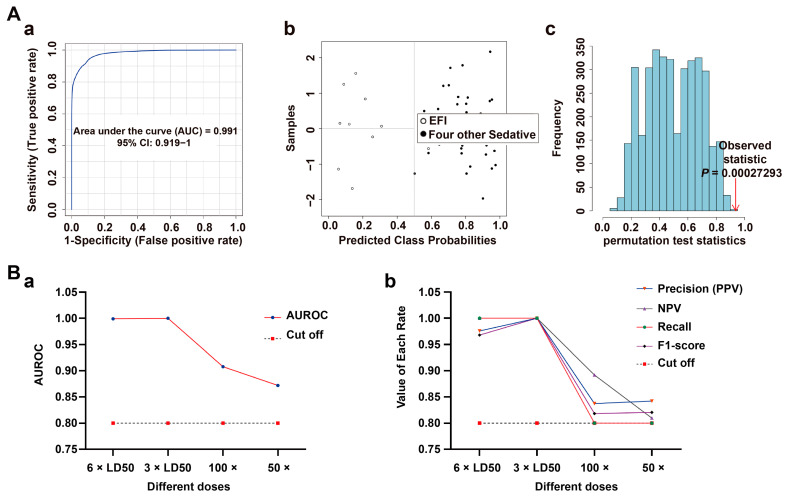
Validation of classification model in brainstem tissue samples. (**A**) Specificity evaluation of the EFI classification model relative to four other sedative-hypnotic drugs’ fatal intoxication models (zaleplon, diazepam, nitrazepam and sodium pentobarbital, n_animal_ = 10, respectively) (**a**) ROC plot: AUC = 0.991 (95% CI: 0.919−1); (**b**) confusion matrix plot showed that one case was wrongly diagnosed as control. (**c**) 100 permutation test plot, *p* < 0.01. (**B**) Sensitivity evaluation of the EFI classification model relative to other different dose groups of estazolam (3 × LD50, 100 × and 50 × therapeutic dose groups, n_animal_ = 10, respectively); (**a**) line plot of AUC-value in different dose groups, cutoff = 0.8. (**b**) Line plot of the Precision (PPV), NPV, Recall and F1-score, cutoff = 0.8.

The classification model was evaluated using the four sedative-hypnotic drugs (Figure 7A(a–c)), and four toxicants’ fatal intoxication models described above, which showed a very high specificity (Figure S2D(a–c)).

Next, three other dose groups (3 × LD50, 100 × and 50 × therapeutic dose intoxication groups) were introduced to validate the sensitivity of the classification model. The variation of the AUC is shown in Figure 7B(a). The AUC (=0.858 and 0.872, respectively) was higher than 0.8 in the two low-dose groups. The line plots of the precision (PPV), NPV, recall and F1 scores of the classification model were consistent with the trend of this result. The recall values of the 100 × and 50 × groups were equal to 0.8, and all other values were greater than 0.8 (Figure 7B(b)). Specific data are shown in Table S10. Thus, the classification model had a high sensitivity.

### 3.5. New Toxicological Mechanism of Estazolam

The overview of global metabolic profiles according to the quantitative results for the EFI- and EIND-group metabolites in the mouse brainstem tissue samples were revealed with PCA scores plots in Figure S3(A). Clustering showed that the two groups partially overlapped, but there were significant differences. PLS-DA analysis was applied to distinguish the two groups (Figure S3(B)).

**Figure 8:**
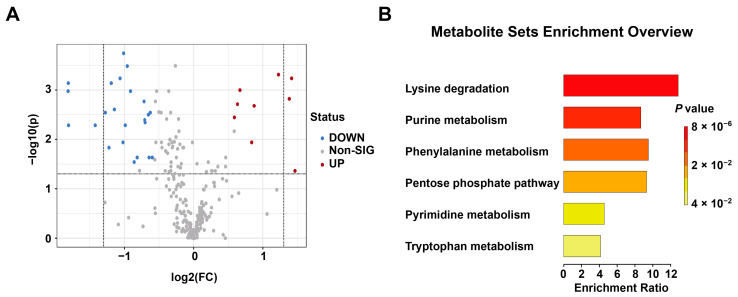
Exploring a new toxicological mechanism of estazolam in brainstem tissue samples between EFI and EIND groups (n_animal_ = 10, respectively). (**A**) Volcano plot of the EFI and EIND group, with nine up-regulated (red points) and 23 down-regulated (blue) differential metabolites. (**B**) In the plot for quantitative enrichment analysis of differential metabolites, the most perturbed pathway is the lysine degradation pathway.

Nine up-regulated and 23 down-regulated differential metabolites were further screened among 244 compounds by *p* < 0.05 and FC > 1.5 or < 0.67, VIP > 1 conditions (Figure 8A). Among them, there were 24 endogenous metabolites, nine were up-regulated and 15 down-regulated. Specific data are shown in Table S11. To screen the differential metabolic pathways between the two groups based on the above differential metabolites, quantitative enrichment analysis was performed, and the results showed that the main differential pathways were lysine degradation, purine metabolism, phenylalanine metabolism, pentose phosphate pathway and pyrimidine metabolism. Among them, the lysine degradation pathway was the most disturbed (Figure 8B). The results related to quantitative enrichment analysis are shown in Table S12.

**Figure 9:**
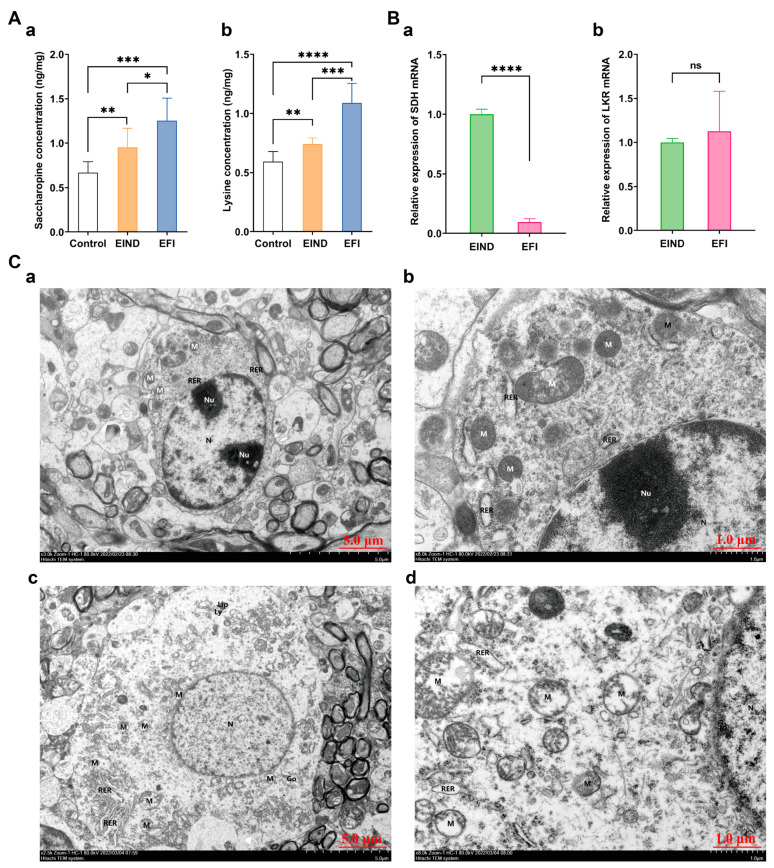
Validation of a new mechanism of estazolam toxicology. (**A**) Quantitative analysis of saccharopine (**a**) and lysine (**b**) between the EFI and EIND and control group (n_animal_ = 10, respectively), which were normalized to the WT (weight, 50mg brainstem tissue) value: *: *p* < 0.05; **: *p* < 0.01; ***: *p* < 0.001; and ****: *p* < 0.0001. (**B**) Histogram of relative expression of SDH and LKR mRNA in two sets of brainstem samples (n_animal_ = 10, respectively): ****: *p* < 0.0001; ns: no statistically significant difference. (**a**) Histogram of relative expression of SDH mRNA between EFI and EIND groups. (**b**) Histogram of relative expression of LKR mRNA between EFI and EIND groups. (**C**) Brainstem ultrastructure between EFI and EIND groups (n_animal_ = 3, respectively); (**a**) brainstem ultrastructure of EIND group, bar = 5 μm; (**b**) EIND group, bar = 1 μm; (**c**) brainstem ultrastructure of EFI group, bar = 5 μm. (**d**) EFI group, bar = 1 μm. (**a**,**b**) The double nucleolus (Nu). The mitochondria (M) were mostly round, some were mildly swollen, individual membranes were broken, the matrix was a little shallow, the cristae were shortened; the rough endoplasmic reticulum (RER) was slightly dilated; (**c**,**d**) The nucleus (N) was oval, mitochondria (M) were mostly round; some of them were moderately to severely swollen, with broken membranes, more matrix lysis, broken and disappearing cristae, and a small amount of vacuolation; the rough endoplasmic reticulum (RER) was locally aggregated and slightly dilated; Golgi apparatus (Go) was slightly hypertrophied; lipofuscin (Lip) and lysosomes (Ly) were present.

Further validation of the lysine degradation pathway revealed that in brainstem samples, the EFI group exhibited a significant increase in saccharopine and lysine concentration than the EIND and control groups, with means for the former of 1.253 ng/mg, 0.9533 ng/mg, and 0.6678 ng/mg, respectively (Figure 9A(a)), and the latter of 1.089 ng/mg, 0.7415 ng/mg, and 0.5914 ng/mg, respectively (Figure 9A(b)), with *p* values < 0.05 for all group comparisons, and the differences were statistically significant. Specific data were shown in Table S13.

Validation with qPCR showed that the relative expression of SDH mRNA was significantly decreased in the EFI group compared to EIND groups, with respective means of 1.206, 2.678, and a statistically significant difference of *p* < 0.05 between the two group’s comparisons. In contrast, there was no statistically significant difference in relative expression of lysine-ketoglutarate reductase (LKR) mRNA, with means of 1.130, and 1.104, respectively (Figure 9B(a,b)).

Finally, the electron microscopy results showed more severe overall damage to mitochondria in the EFI group. Some mitochondria showed moderate to severe swelling, broken membranes, more matrix lysis, broken cristae, and a few vacuoles (Figure 9C(c,d)). In contrast, mitochondria in the EIND group were less damaged; most had fine structure and uniform matrix, with a small amount of mild swelling, and small numbers of broken membranes and simple matrixes (Figure 9C(a,b)). Significant differences in mitochondrial damage existed between the two sample groups.

## 4. Discussion

In recent years, fatal intoxication by sedative-hypnotic drugs had steadily increased [4,5]. There are, however, no systematic data on the plasma concentrations of fatal intoxication with such drugs [13]. There is even an overlap of plasma concentration values between the fatal and intoxication groups [9]. Therefore, it is typically challenging to use the plasma concentration as an accurate indicator when identifying deaths resulting from this type of iatrogenic lethal poisoning, particularly in complex instances. In this study, considering that one branch of metabolomics, thanatometabolomics [16], can perform a metabolomic autopsy and potentially screen biomarkers [15], this approach was applied to construct a classification model (in plasma and brainstem samples, respectively) for estazolam fatal intoxication to provide molecular evidence [17] for its cause-of-death identification, based on which the toxicological mechanism of estazolam was further explored. Eventually, a more scientific and exact identification approach was developed to identify the cause of death in fatal estazolam intoxication and a new understanding of this drug’s intoxication mechanism.

To observe the overlap of plasma concentrations mentioned in the above literature, this study analyzed the plasma concentration data of two groups (EFI and EIND) of mice with the same dose and dosing time, but with different results. Moreover, it was found that the plasma concentration ranges of estazolam’s fatal intoxication and non-death intoxication groups not only had a large overlap interval but also differed significantly between groups (Figure 1). This result was consistent with the previous literature [9] in that the plasma concentration values were not an objective criterion. Thus, a more accurate identification method was needed.

Therefore, we set up four negative control groups (NDRDs) to compare with the EFI mouse models to establish highly predictive classification models, and conducted the metabolomic analysis. These control groups were set up because they had a common influencing factor, which was hypoxia, and to increase the specificity of the differential metabolites. The metabolic profiles of the EFI and the four NDRD groups can be observed in Figure 2 and Figure 5. The EFI group was completely separated from the four NDRD groups without overlap. A biomarker meta-analysis approach was used to screen the EFI group for common differential metabolites with each NDRD group; as there were more than four data sets, a more visualized upset plot was used to facilitate their reading and interpretation [40].

After obtaining the common differential metabolites, further screening was performed to obtain the candidate differential metabolites. The application of a biomarker analysis combined with a multivariate ROC curve-based test analysis using the linear SVM algorithm was applied to develop a classification model for estazolam fatal intoxication. Simple models consisting of a few biomarkers are preferred over the complex models that include many biomarkers, because they are more robust, cost-effective, and easier to fit well [35]. A discriminative classification model for EFI was found in plasma based on three possible differential metabolites, phenylacetylglycine, creatine, and indole-3-lactic acid. A discriminatory model was created in brainstem tissue based on palmitic acid, creatine, and indole-3-lactic acid. However, each selected endogenous compound was not a differential metabolite unique to estazolam’s fatal intoxication [41,42,43,44]. However, the combination of several of the above-mentioned endogenous compounds were unique to the drug.

After the model was constructed, its predictive power needed further evaluation in terms of discriminability and reliability. Based on this signal detection framework, measures for evaluating reliability (PPV and NPV) may be retrieved from the ROC curve, an important discriminability tool [36]. Based on this, there are almost no misclassified samples when the AUC values of the classification models are equal to 1 or very close to 1; then the PPV and NPV values are equal to or close to 1 [45]. The ROC curve and confusion matrix plot can visualize the predictive power of the classification model. However, interpreting the confusion matrix becomes difficult when the class probability decreases and the misclassified samples increase. Therefore, based on the rich information of the confusion matrix plot [46], this study combined the above reliability evaluation and machine learning evaluation metrics: Precision (PPV), NPV, Recall, and F1-score to evaluate the model comprehensively [36,37,47]. As shown in Figure 3 and Figure 5, when comparing the EFI group with the four sedative-hypnotics and the four toxicants, all the above indicators were high (equal to 1 or very close to 1), and the two sets of classification models were highly discriminatory and reliable, with high specificity. Moreover, the models’ sensitivity was very high when the 3 × LD50 group and the 50 × and 100 × therapeutic dose intoxication groups could be distinguished from the control group. Then in forensic practice, the classification model can be applied to intoxication cases with tens or even hundreds of times the therapeutic dose of this drug [48].

Furthermore, concerning the stability of plasma samples for 0–20 days, this study showed that the predictive ability of the classification model was very strong at all six-time points (0, 1, 5, 10, 15, and 20 days) within 20 days. However, the Recall, Precision (PPV), and F1-score at the last time point were slightly below 0.9. Thus, the classification model proved robust for detecting this drug-induced fatal overdose within 15 days of postmortem examination [49].

Here, we return to the plasma concentration values overlapping between the EFI and EIND groups to investigate their causes. The results of quantitative metabolic pathway enrichment analysis of the differential metabolites of these two groups showed that the lysine degradation metabolic pathway was most strongly perturbed. This lysine degradation pathway is potentially responsible in estazolam’s fatal intoxication. According to the literature, α-aminohemialdehyde synthase (AASS), a key enzyme in the lysine degradation pathway, is a bifunctional enzyme with a lysine-ketoglutarate reductase (LKR) structural domain at the N-terminal end and a saccharopine dehydrogenase (SDH) structural domain at the C-terminal end. Numerous reasons, including mutations in AASS, cam cause a decrease, or even a loss, of the catalytic function of the saccharopine dehydrogenase (SDH) structural domain at the C-terminal end, which in turn leads to the accumulation of saccharopine [50], an intermediate product of lysine degradation, which in mitochondria causes abnormalities in their morphology, structure and function, thus affecting the normal energy metabolism of tissues and organs [51].

As shown in Figure 9, qPCR validation showed that the relative expression of SDH was lower in the EFI group than in the EIND and control groups, with statistically significant differences. Estazolam’s toxic effects superimposed on saccharopine accumulation and mitochondrial dysfunction caused by the decreased catalytic activity of SDH, a key enzyme of the lysine degradation pathway, led to central respiratory inhibition. In contrast, mice in the EIND group without superimposed aberrant lysine degradation showed only drug toxicity and did not die. This result was supported by the quantification of brainstem lysine and saccharopine. Additional electron microscopy findings supported more mitochondrial damage in the EFI group compared to the EIND group [51]. This result further supports the association of estazolam’s fatal intoxication with abnormal lysine degradation pathways.

In this study, two sets of discriminative classification models with high discriminability and reliability for estazolam fatal intoxication were constructed by combining MS/MS full-scan coupled with metabolomic approaches in plasma and brainstem tissue samples, respectively. The sensitivity and specificity of the above classification models were evaluated and validated in different categories of sedative-hypnotic drugs and different dose-groups of estazolam, respectively, and the stability of the classification model was validated in plasma samples. We also analyzed and validated estazolam’s fatal intoxication and concluded that the lysine degradation pathway is an important synergistic site for its toxic effects. This provides a new idea for the identification and mechanism study of estazolam’s fatal intoxication.

There were a few limitations in our study that should not be ignored. First, estazolam’s fatal intoxication was only validated in the lysine degradation pathway; other metabolic pathways need further validation. Second, the discriminative classification model developed in this study was not realistically verified on human specimens due to a shortage of such specimens. We hope to collaborate with other forensic identification institutes to gather equivalent specimens.

## Figures and Tables

**Table 1:** Differential metabolites for estazolam fatal intoxication in plasma samples.

Compound Name ^a^	Metabolite Identification			*p* ^d^	Combined Log FC ^e^	Trend ^f^	Combined VIP ^g^
	Accurate Mass ^b^	Retention Time ^b^	mzCloud Best Match ^c^				
Phenylacetylglycine	193.07388	6.414	90	1.52 × 10^−33^	1.822502209	up	1.27002
7-Methylguanine	165.06493	1.767	87.1	1.26 × 10^−32^	1.812534031	up	1.03212
Propionylcarnitine	217.13116	2.578	92.9	9.31 × 10^−32^	−1.80225988	down	1.09656
Creatine	131.06937	1.489	95.2	4.95 × 10^−30^	1.780471987	up	1.2083
L-Ascorbic acid 2-sulfate	255.98905	1.222	88	4.22 × 10^−28^	1.754421741	up	1.07764
Methionine	149.0509	1.67	91.8	3.98 × 10^−25^	−1.706198279	down	1.27548
Ascorbic acid	176.03203	1.226	87.4	1.54 × 10^−23^	1.676402203	up	1.11066
N-Isovalerylglycine	159.08942	5.848	89.3	3.21 × 10^−22^	1.649229414	up	1.1655
4-Pyridoxic acid	183.05305	3.268	94.8	1.91 × 10^−21^	1.632156605	up	1.16982
Xanthurenic acid	205.03731	5.402	91.2	6.65 × 10^−21^	1.619327608	up	1.25248
Acetyl-L-carnitine	203.11558	1.659	93.8	4.74 × 10^−20^	−1.599262594	down	1.09352
Indole-3-lactic acid	205.07359	6.928	91.8	1.62 × 10^−18^	1.560302287	up	1.060518
Valine	117.0788	1.619	95.8	8.78 × 10^−15^	−1.443708344	down	1.004272
3-Phenyllactic acid	166.06286	6.78	91.1	5.48 × 10^−7^	1.036145258	up	1.18646

^a^: The annotation for each compound was based on the HMDB database; ^b^: accurate mass and retention time were obtained from the Q Exactive mass spectrometer on a full-scan model; ^c^: the mass spectrometry data were analyzed by CD 3.1 software (version, Manufacturer name, city, state abbreviation if US or Canada, country) to obtain; ^d^: calculated with student’s *t*-test. *p* < 0.05 means that differences in this metabolite between EFI and the controls are significant; ^e^: Combined Log FC: in the biomarker meta-analysis, the mean of fold-change for the metabolite in the experimental group relative to the different control groups is one of the main bases for screening the common differential metabolites; ^f^: trend compared to the control group; ^g^: combined VIP: this value represents the ability of each metabolite to explain estazolam fatal intoxication, and is the mean value of the VIP-value of the experimental group and each control group and auxiliary screening for differential metabolites (the usual screening criterium is VIP > 1).

**Table 2:** The diagnostic efficacy of various metabolites in plasma samples.

Compound Name	AUC	95%CI	*p*
Phenylacetylglycine	1	1−1	11.3668 × 10^−15^
Creatine	0.9975	0.989−1	3.4137 × 10^−15^
Indole-3-lactic acid	0.96	0.894−1	1.5252 × 10^−10^

**Table 3:** Differential metabolites for estazolam fatal intoxication in brainstem tissue samples.

Compound Name	Metabolite Identification			*p*	Combined Log FC	Trend	Combined VIP
	Accurate Mass	Retention Time	mzCloud Best Match				
Palmitic acid	256.23977	9.512	96.3	2.09 × 10^−21^	−1.653094773	down	1.63824
Prostaglandin D2	352.22435	8.491	93.1	3.22 × 10^−21^	−1.648333391	down	1.8836
Indole-3-lactic acid	205.07668	6.625	89.6	7.08 × 10^−21^	1.640230298	up	1.72006
Creatine	131.06942	1.05	96.7	2.39 × 10^−15^	−1.48559731	down	1.24968
DL-Tryptophan	204.08967	5.174	87.3	2.49 × 10^−15^	1.483820281	up	1.48568
Indole-3-acrylic acid	187.06325	5.176	93.4	3.98 × 10^−15^	1.476502537	up	1.48258

**Table 4:** The diagnostic efficacy of various metabolites in brainstem tissue samples.

Compound Name	AUC	95%CI	*p*
Palmitic acid	0.98	0.939−1	9.6901 × 10^−11^
Indole-3-lactic acid	0.973	0.914−1	1.6272 × 10^−9^
Creatine	0.958	0.896−0.992	3.0926 × 10^−7^

## Data Availability

The data presented in this study are available in Supplementary Material.

## Supplementary Materials

The following supporting information can be downloaded at: https://www.mdpi.com/article/10.3390/metabo13040567/s1. Figure S1: Evaluation of PCA and PLS-DA models for plasma samples and the results of EFI classification model validation; Figure S2: evaluation of PCA and PLS-DA models for brainstem samples and the results of EFI classification model validation; Figure S3: overview of metabolic profiles of the EFI and EIND groups. Table S1: summary of group information; Table S2: metabolite classes and proportions in plasma and brainstem tissue samples; Table S3: AUC values of different candidate differential metabolite combinations in plasma samples; Table S4: prediction results of new plasma samples (validation set); Table S5: relative blood drug concentration of estazolam in different dose groups (plasma samples); Table S6: sensitivity validation results of classification models in estazolam different dose groups (plasma samples); Table S7: stability validation results of classification models in different storage time groups (plasma samples); Table S8: AUC values of different candidate differential metabolite combinations in brainstem tissue samples; Table S9: prediction results of new brainstem samples (validation set); Table S10: sensitivity validation results of classification models in estazolam different dose groups (brainstem samples); Table S11: endogenous differential metabolites in the EFI group vs the EIND group; Table S12: results from quantitative enrichment analysis of EFI group vs EIND group; Table S13: quantification of metabolites in brainstem tissue samples (ng/mg).
