# Supplementary material for: A Metabolomics-Based Study on the Discriminative Classification Models and Toxicological Mechanism of Estazolam Fatal Intoxication
Source: Metabolites. 2023 Apr 17;13(4):567. doi: 10.3390/metabo13040567 (PMC10144813; doi:10.3390/metabo13040567)
Supplement: Supplementary file 1 [file metabolites-13-00567-s001.zip › metabolites-2287723-supplementary.pdf]

Supplementary Material

S1. Materials and Methods

Table S1. Summary of group information.

Groups	Drug (Dose)	Mice					Aim
		Number	Age	Sex	Treatments	Sample Type	
EFI & Control (four NDRDs (CD, MA, DR and HS))	EFI: 500 mg/100g (6 × LD50)<br>NDRDs: non-drug related deaths	10, respectively	7–8 weeks	Female: Male = 1:1	EFI: Death from drug poisoning<br>The DR, MA and HS groups were given cervical dislocation at the time of almost dying.	Plasma, Brainstem	Differential metabolites screening & Classification model construction
EFI & New Control (four NDRDs (CD, MA, DR and HS))	EFI: 500 mg/100g (6 × LD50)	10, respectively	7–8 weeks	Female: Male = 1:1	ibid	Plasma	Testing of classification model predictive power in validation set
EFI & Control (three NDRDs (CD, MA, DR))	EFI: 500 mg/100g (6 × LD50)	EFI: 8; (CD, DR, MA, *n* = 6, respectively)	7–8 weeks	Female: Male = 1:1	ibid	Plasma	Stability analysis of Classification Model within 20 days.
EFI & Four other sedative-hypnotics fatal intoxication	EFI: 500 mg/100g (6 × LD50)<br>diazepam: 280 mg/100g (5 × LD50)<br>nitrazepam: 275 mg/100g (5 × LD50)<br>zaleplon: 280 mg/100g (Same as diazepam)<br>sodium pentobarbital：20 mg/100 g (≈ 1.6 × LD50)	10, respectively	7–8 weeks	Female: Male = 1:1	Death from drug poisoning	Plasma, Brainstem	Specificity evaluation of the Classification Model
EFI & Four other toxicants fatal intoxication	EFI: 500 mg/100g (6 × LD50)<br>Heroin: 21.8 mg/100g<br>DL-METH: 43.2 mg/100 g<br>CO/CO2: Replace the anesthesia box volume at a rate of 10-30% per minute into the box, ≈2 min	10, respectively	7–8 weeks	Female: Male = 1:1	Death from drug poisoning<br>Death from toxicant poisoning	Plasma, Brainstem	Specificity evaluation of the Classification Model
EFI & Three other different doses of Estazolam intoxication	EFI: 500 mg/100g (6 × LD50)<br>EFI: 250 mg/100g (3 × LD50)<br>50 x Estazolam therapeutic: 2.59 mg/100 g<br>100 x Estazolam therapeutic: 5.17 mg/100 g	10, respectively	7–8 weeks	Female: Male = 1:1	Death from drug poisoning<br>Administered for 24 h, euthanized by cervical dislocation	Plasma, Brainstem	Sensitivity evaluation of the Classification Model
EFI & EIND	EFI/EIND: 500 mg/100g (6 × LD50)	10, respectively	7–8 weeks	Female: Male = 1:1	EFI: Death from drug poisoning	Brainstem	Toxicological mechanism analysis and validation (QEA & qPCR & TEM)
EFI & EIND & Control (CD)	EFI/EIND: 500 mg/100g (6 × LD50)	10, respectively	7–8 weeks	Female: Male = 1:1	EIND:After administration not dead for more than 8 h and treated with cervical dislocation	Brainstem	Toxicological mechanism validation (Metabolite Quantification)

S1.1. Experimental Design

Plasma concentration is not an uncontested criterion for identifying fatal estazolam intoxication as the cause of death. Estazolam fatal intoxication and intoxication non-death plasma concentration data demonstrated this result (quantified by UPLC (ExionLCTM AC, SCIEX, Framingham, MA, USA) in conjunction with a 5500 Q-Trap system (SCIEX) in multiple reaction monitoring modes). Therefore, a non-targeted metabolomics approach was introduced to construct a classification model for the fatal intoxication caused by this drug to determine the death cause and explore its new toxicological mechanism. There are four sets of animal models of estazolam fatal intoxication and intoxication at different doses. Four sets of non-drug intoxication death groups associated with hypoxia (control) and four sets of other fatal intoxication poisons. The other drugs and toxicants fatal intoxication mice models were prepared by intragastric administration except for gas intoxication. After a series of treatments, plasma samples from the abdominal aorta and brainstem tissue samples from each mouse group were analyzed by liquid chromatography-high resolution tandem mass spectrometry (LC-HR MS/MS) in full scan mode. The instrument utilized in this step is a UPLC (Ultimate 3000) combination of a Q Exactive mass spectrometer. The raw data from the LC-HR MS/MS were further processed with Compound Discoverer 3.1. Those metabolites with MS2 information were

subjected to multivariate analysis with MetaboAnalyst 5.0 to complete screening of potential differential metabolite and the classification model construction between estazolam fatal intoxication group and four sets of non-drug intoxication death groups for plasma samples and brain stem tissue samples. The specificity of the classification model was verified in the lethal group of four other poisons and four sedative-hypnotic drugs, and the sensitivity of the classification model was verified in the different dose groups of estazolam. The classification model's stability was also verified in the blood samples. Finally, metabolic pathway enrichment analysis was conducted in the groups of estazolam fatal intoxication and not death, and the most disturbed differential metabolic pathway was obtained. This pathway was further verified to explore the new toxicological mechanism of estazolam.

S1.2. Chemicals and Reagents

Heroin and DL-METH were provided by Beijing Municipal Public Security Bureau (Beijing, China). Carbon monoxide (liquefaction) and carbon dioxide (liquefaction) were purchased from Shijiazhuang Xisanjiao Practical Gas Co. LTD (Shijiazhuang, China).

S1.3. Animals Diets and Grouping

Subsequently, to assess the specificity of the classification model, four other toxicant fatal intoxication models ($n = 10$, respectively, F: M = 1:1) were introduced; namely, Heroin (21.8 mg/100g), DL-METH (43.2 mg/100g), Acute carbon monoxide fatal intoxication model and Acute carbon dioxide fatal intoxication model, CO and CO2 models were made according to the euthanasia treatment of mice.

S1.4. Determination of Plasma Concentration

In total, 200 μL whole blood samples after thawing at 4 °C were added to tubes with 790 μL ice-cold extractant ($V_{acetonitrile}$: $V_{Milli\text{-}Q\ water}$ = 3:1), and 10 μL SKF525A (100 ug/mL) was used as an internal standard. Another blank plasma was mixed with different volumes of 1 mg/mL of estazolam stock solution (dissolved in methanol) to configure 1 mL of 250, 500, 1000, and 2000 μg/mL of estazolam plasma, then 200 μL of which was taken into 790 mL of ice-cold extractant and 10 μL of internal standard working solution as described above to configure a series of the calibration curve (50, 100, 200, 400 μg/mL of estazolam) for quantification Each sample was then vortexed for 30 s, sonicated for 10 min in an ice-water bath, and incubated for 20 min at −20 °C to allow protein precipitation. The mixtures were centrifuged at 12,000 g for 10 min at 4 °C. The resulting supernatants diluted 100 times with methanol were transferred to HPLC vials for LC-MS/MS analysis.

S1.5. Multiple Reaction Monitoring (MRM) Analysis by LC-MS/MS

The LC-MS/MS system was an ExionLC™ AD (AB SCIEX, Framingham, MA, USA) coupled with a SCIEX QTRAP 5500 triple quadrupole mass spectrometry (AB SCIEX) with an ESI source used for separation and detection the plasma concentration of estazolam. The column used for this study was a Kinetex C18 column (2.1 × 100 mm, 2.6 μm; Phenomenex, Torrance, CA, USA). The column temperature was maintained at 40 °C. The elution gradient with a gradient elution of mobile phase A (1 mmol/L ammonium acetate with 0.01% formic acid in Milli-Q water) and mobile phase B (acetonitrile) at a flow rate of 0.4 mL/min for 12 min. and an injection volume of 5 μL. The elution gradient was set as follows: 0 min, 97% A, 0.5 min, 97% A; 9 min, 5% A; 10 min, 5% A; 10.1 min, 97% A;12 min, 97% A. The Mass spectrometry was performed in positive electrospray ionization utilizing multiple reaction monitoring (MRM) modes. The source-dependent parameters were as follows: ion spray voltage 5500 V, curtain gas 30 psi, vaporizer temperature 550 °C, nebulizing gas (GS1) 60 psi, and drying gas (GS2) 70 psi. Data acquisition and processing were performed using Analyst software version 1.6 (SCIEX).

S1.6. Saccharopine and Lysine Measurement

To measure saccharopine and lysine levels in mice brainstems, 50 mg of brain stem tissue (n_{EFI} = n_{EIND} = 10, F:M = 1:1) was thawed at 4 °C mixed with 500 μL of ice-cold methanol. An appropriate amount of grinding beads was added and ground at −4 °C for 90 s to form a homogenized slurry. Then, it vortexed for 30 s, sonicated for 10 min in an ice-water bath, and incubated for 20 min at −20 °C to allow protein precipitation. The mixtures were then centrifuged at 12,000 g for 10 min at 4 °C, and the supernatant was collected and evaporated to dryness under nitrogen and redissolved with 50 μL of methanol containing SKF525A (10 ng/mL). Since a plasma sample devoid of these two standards is unavailable, methanol calibration curves were prepared. Briefly, 1 mg/mL of lysine and saccharin stock solution was serially diluted to generate the calibration curve. A 495 μL aliquot of the appropriate dilution was transferred into a chromatographic tube and mixed with 5 μL internal standard-SKF525A (1 μg/mL) for each point. All samples were stored at 4 °C for LC-MS/MS analysis. In the LC-MS/MS system as above, the column used for this part was a Luna PFP (2) column (4.6 × 150 mm, 5 μm; Phenomenex, Torrance, CA, USA). The column temperature and mobile phase are consistent with the previous. The flow rate was 0.3 mL/min, and an injection volume of 2 μL. The elution gradient was set as follows: 0 min, 98% A; 1 min, 98% A; 5 min, 2% A; 8 min, 2% A; 8.1 min, 98% A; and 10 min, 98% A. The Mass spectrometry was performed in positive electrospray ionization utilizing multiple reaction monitoring (MRM) modes. The source-dependent parameters were as follows: ion spray voltage 5500 V, curtain gas 30 psi, vaporizer temperature 550 °C, nebulizing gas (GS1) 55 psi, and drying gas (GS2) 55 psi. Data acquisition and processing were performed using Analyst software version 1.6 (SCIEX).

S1.7. Quantitative Real-time PCR

RNA was isolated from brainstem tissues (n_{EFI} = n_{EIND} = 10, F:M = 1:1) after low-temperature grinding using a Trizol. PrimeScript RT reagent Kit (Takara) was used to reverse-transcribe RNA in a 20-μl reaction mixture. Quantifying SDH and LKR mRNA expression was performed using the TB Green PremixEx TaqTM II kit (Takara) by a real-time PCR system (QuantStudio 7 Flex, Applied Biosystems, Waltham, MA, USA) in quadruplicate. Gapdh was amplified as the internal control. The data were analyzed by the comparative Ct method ($2^{-\Delta\Delta Ct}$). The gene sequences of the primers are as follows:

AASS-LKR forward primer: 5′-AGGGTCTCGGATAGTGGCTTTCG-3′,
AASS-LKR reverse primer: 5′-GGCTGCTGTTCCTGTAGTTGTGAG-3′,
AASS-SDH forward primer: 5′-CCACCACAGGATGAAGCACATAAGG-3′,
AASS-SDH reverse primer: 5′-GTTGGCAAGCAAGAGGCAAAGC-3′.

S1.8. TEM Analysis

After the mice in the EFI group died, and the mice in the EIND and control groups were euthanized by cervical dislocation, the brain stem tissue was taken immediately within 1–3 min. A sharp blade was used to cut and harvest fresh tissue blocks（no more than 1 mm^3) to minimize mechanical damage. The small tissue pieces were then immediately transferred to Petri dishes on ice in ice-cold fresh TEM fixative to trim the size and then transferred into EP tubes with ice-cold fresh fixative for further fixation for 24 h. Then the tissues were washed thrice with 0.1 M PB (pH 7.4) for 15 min each. Then the samples were washed with phosphate buffer thrice (5 min for each time). Tissues avoid light post-fixed with 1% OsO_4 in 0.1 M PB (pH 7.4) for 2 h at room temperature. After removing OsO4, the tissues are rinsed in 0.1 M PB (pH 7.4) thrice for 15 min each. Dehydration at room temperature was performed by a series of graded ethanol solutions (30, 50, 70, 80, and 95% ethanol solutions) for 20 min for each step, and the 100% ethanol step was performed twice (20 min for each time), and a 100% acetone step twice more (15 min each). Then samples were infiltrated and embedded by acetone and embedding media 812 with different proportions. After a series of resin embedding procedures at 37 °C for approximately 36 h, it transferred to 65 °C to polymerize for more than 48 h. Sections were cut by ultra-microtome into 60–80 nm and stained with uranyl acetate and

lead citrate to capture images with the transmission electron microscope (HT7800, Hitachi, Tokyo, Japan).

S2. Results

Table S2. Metabolite classes and proportions in plasma and brainstem tissue samples

Class (HMDB)	Metabolites					
	Plasma			Brainstem		
	Number	Percentage		Number	Percentage	
Organic acids and derivatives	106	30.11%		78	35.78%	
Lipids and lipid-like molecules	101	28.69%		47	21.56%	
Organoheterocyclic compounds	49	13.92%		37	16.97%	
Benzenoids	40	11.36%		21	9.63%	
Organic oxygen compounds	22	6.25%	89.80%	10	4.59%	89.34%
Nucleosides, nucleotides, and analogues	12	3.41%		10	4.59%	
Alkaloids and derivatives	7	1.99%		0	0	
Organic nitrogen compounds	7	1.99%		0	0	
Others	8	2.27%		15	6.88%	
No-class	40		10.20%	26		10.66%
Total	392			244		

S2.1. A Brief Procedure for Screening the Constituent Components of the EFI Classification Models

During the screening we selected the combinations of candidate differential metabolites in Table 1 that had relatively large effects on the EFI group, using the values of *P* value, Combined Log FC, et al. as criteria for selection. In addition, metabolites that were stably present in several pre-experiments were only included. In order to construct a classification model with better discriminatory power, we controlled the inclusion criteria for the modeling components more strictly than the common differential metabolites screening.

The ROC curve analysis (in the training set) based on SVM algorithm was first performed with five compounds combinations, and next it was found that the combination of four compounds could also achieve an AUC value of 1. When candidate differential metabolites were gradually included in order of importance, the AUC value of combination 7 decreased, then "Valine" was deleted. Three of the remaining six candidate differential metabolites were randomly selected for recombination, and then ROC curve analysis was done. Four combinations all had an AUC value of 1. The ROC test was further continued in a new set of samples, and combination 9 had an AUC value of 1 under all algorithms, with higher predictive power than the remaining three combinations. The three candidate differential metabolites of combination 9 (creatine, phenylacetylglycine, and indole-3-lactic acid) were finally identified as the constituent components of the classification model. For details, please refer to the results in Table S3 below. The construction of the classification model in brainstem tissues had the experience of screening in plasma samples. We directly used three candidate differential metabolites to constitute different combinations. Then the AUC curve analysis was performed, the process that was more concise than in plasma samples. As shown in the results of Table S8 (Prostaglandin D2 and DL-Tryptophan were not included because they could not be identified in each batch of brainstem samples) in the Supplementary Material and will not be repeated here.

Table S3. AUC values of different candidate differential metabolite combinations in plasma samples

Candidate differential metabolites	Combinations of different candidate differential metabolites										
Combined Log FC > 1, or < -1, $P < 0.05$, mzCloud Best Match > 90	1	2	3	4	5	6	7	8	9	10	11
Phenylacetylglycine	√			√				√	√	√	√
Creatine	√	√		√	√			√	√	√	√
Methionine	√	√	√	√	√	√		√			
Xanthurenic acid	√	√	√	√	√	√	√			√	
Acetyl-L-carnitine	√	√	√		√	√	√				√
Indole-3-lactic acid		√	√			√	√		√		
Valine			√				√				
**AUC-value**	1	1	1	1	1	1	0.999	1	1	1	1
**AUC-value (new samples)**								0.999	1	0.996	0.998

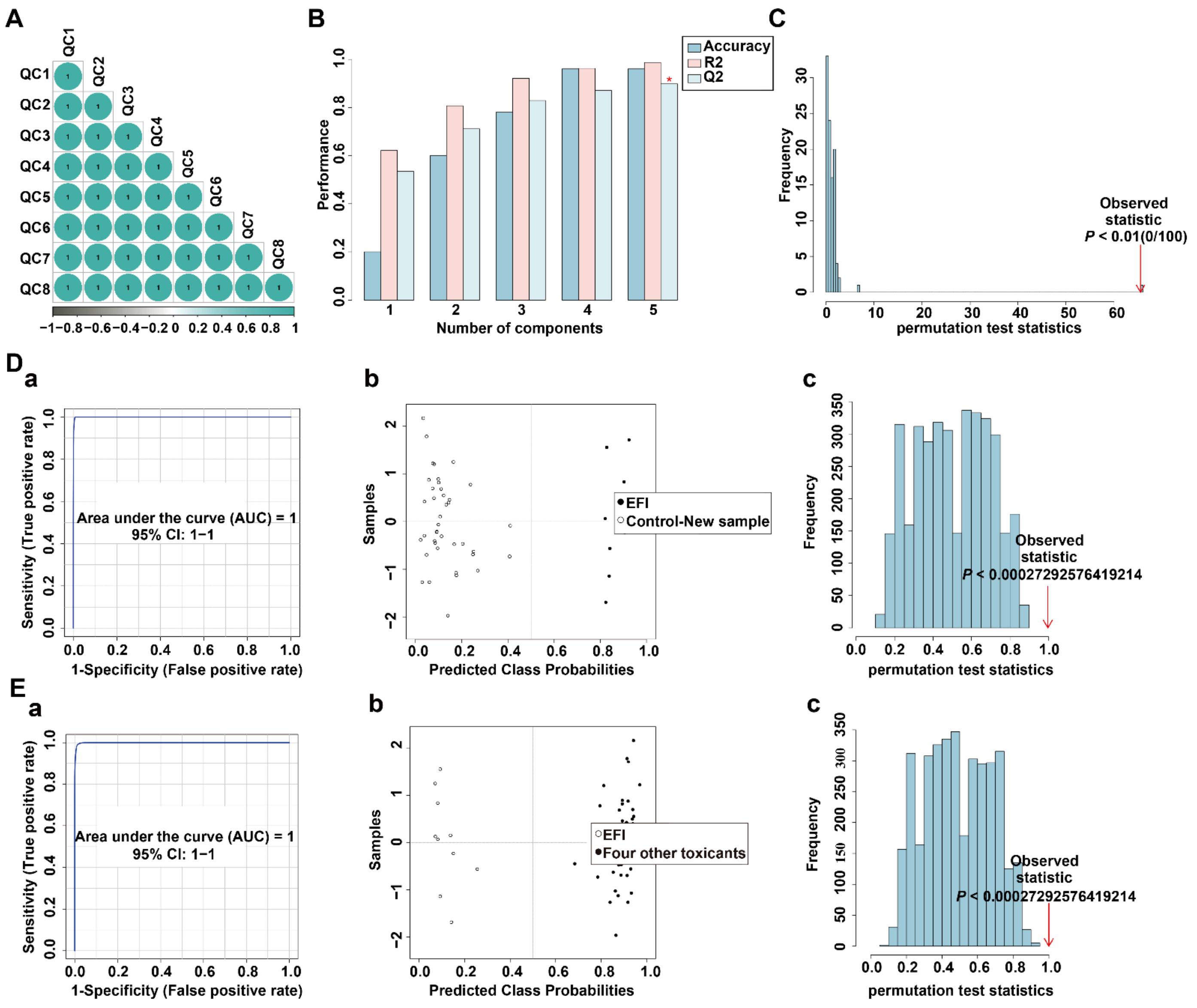

Figure S1. Evaluation of PCA and PLS-DA models for plasma samples and the results of EFI classification model validation. A: Correlation analysis plot of QC samples. B: PLS-DA model cross-validation plot by 10-fold CV algorithm, the five-component model was the best (marked ∗), R2 =

0.98675 and Q2 = 0.89801. C: PLS-DA model with 100 permutation-test plots, p < 0.01. D: Discriminatory ability evaluation in the test set ($n_{EFI, CD, DR, MA, HS}$ = 10, respectively). (a) ROC plot: AUC = 1 (95% CI: 1–1) (b) Confusion matrix plot showing samples without misclassification. (c) Classification model with 100 permutation-test plots, p < 0.01. E: Predictive performance of classification models in the EFI group relative to the four toxicants fatal intoxication models (Heroin, DL-METH, CO, CO2, n_{animal} = 10 respectively) in plasma samples. (a) The ROC plot shows that the AUROC value is 1 (95% CI: 1–1) (b) Confusion matrix plot showing samples without misclassification. (c) Classification model with 100 times permutation test plot, p < 0.01.

To further verify the Specificity of this classification model, four more toxicant lethal models (Heroin, DL-METH, CO, CO_2) in plasma samples were introduced for the above ROC testing. The results of the ROC plot (AUC = 1) and the distribution states of the confusion matrix plot showed that the classification model could well distinguish the EFI group from the other four toxic poisoning lethal groups, and the result of the 100 times permutation test (P < 0.01) showed that the model had good predictive power. The model has good predictive power and strong specificity. (Figures S1Ea–c)

Table S4. Prediction results of new plasma samples (Validation set).

Sample No.	Probability	Class
**1**	0.94691	EFI
**2**	0.94093	EFI
**3**	0.99081	Control
**4**	0.97394	Control
**5**	0.99239	Control
**6**	0.96297	Control
**7**	0.99167	Control
**8**	0.97816	Control
**9**	0.93148	Control
**10**	0.93038	Control

Table S5. Relative blood drug concentration of estazolam in different dose groups (plasma samples).

No.	6 × LD50	3 × LD50	100 ×	50 ×
1	8.84×10^{10}	6.70×10^{10}	1.66×10^{7}	2.45×10^{7}
2	9.12×10^{10}	7.91×10^{10}	1.63×10^{7}	7.58×10^{6}
3	7.75×10^{10}	7.56×10^{10}	2.46×10^{7}	4.80×10^{7}
4	8.30×10^{10}	8.31×10^{10}	1.99×10^{7}	1.62×10^{7}
5	8.51×10^{10}	7.19×10^{10}	1.80×10^{7}	1.98×10^{7}
6	8.02×10^{10}	7.15×10^{10}	1.16×10^{7}	9.47×10^{6}
7	9.08×10^{10}	5.94×10^{10}	5.28×10^{7}	1.32×10^{7}
8	7.38×10^{10}	6.27×10^{10}	7.38×10^{6}	1.66×10^{7}
9	9.14×10^{10}	6.62×10^{10}	1.41×10^{7}	1.00×10^{7}
10	1.00×10^{11}	6.86×10^{10}	2.16×10^{7}	1.49×10^{7}

Table S6. Sensitivity validation results of classification models in estazolam different dose groups (plasma samples).

	6 × LD50	3 × LD50	100 ×	50 ×
TP	10	10	8	8
TN	40	40	32	35
FP	0	0	8	5
FN	0	0	2	2
**Precision (PPV)**	1	1	0.8	0.8649
**NPV**	1	1	0.8	0.8140
**Recall**	1	1	0.8	0.8000
**F1-score**	1	1	0.8	0.8386

Table S7. Stability validation results of classification models in different storage time groups (plasma samples).

	0day	1day	5day	10day	15day	20day
**AUC-value**	1	1	0.998	0.991	0.994	0.971
TP	8	8	8	8	8	7
TN	18	18	17	17	17	16
FP	0	0	1	1	1	2
FN	0	0	0	0	0	1
**Precision (PPV)**	1.0000	1.0000	0.9474	0.9474	0.9474	0.8873
**NPV**	1.0000	1.0000	1.0000	1.0000	1.0000	0.9412
**Recall**	1.0000	1.0000	1.0000	1.0000	1.0000	0.8750
**F1-score**	1.0000	1.0000	0.9730	0.9730	0.9730	0.8811

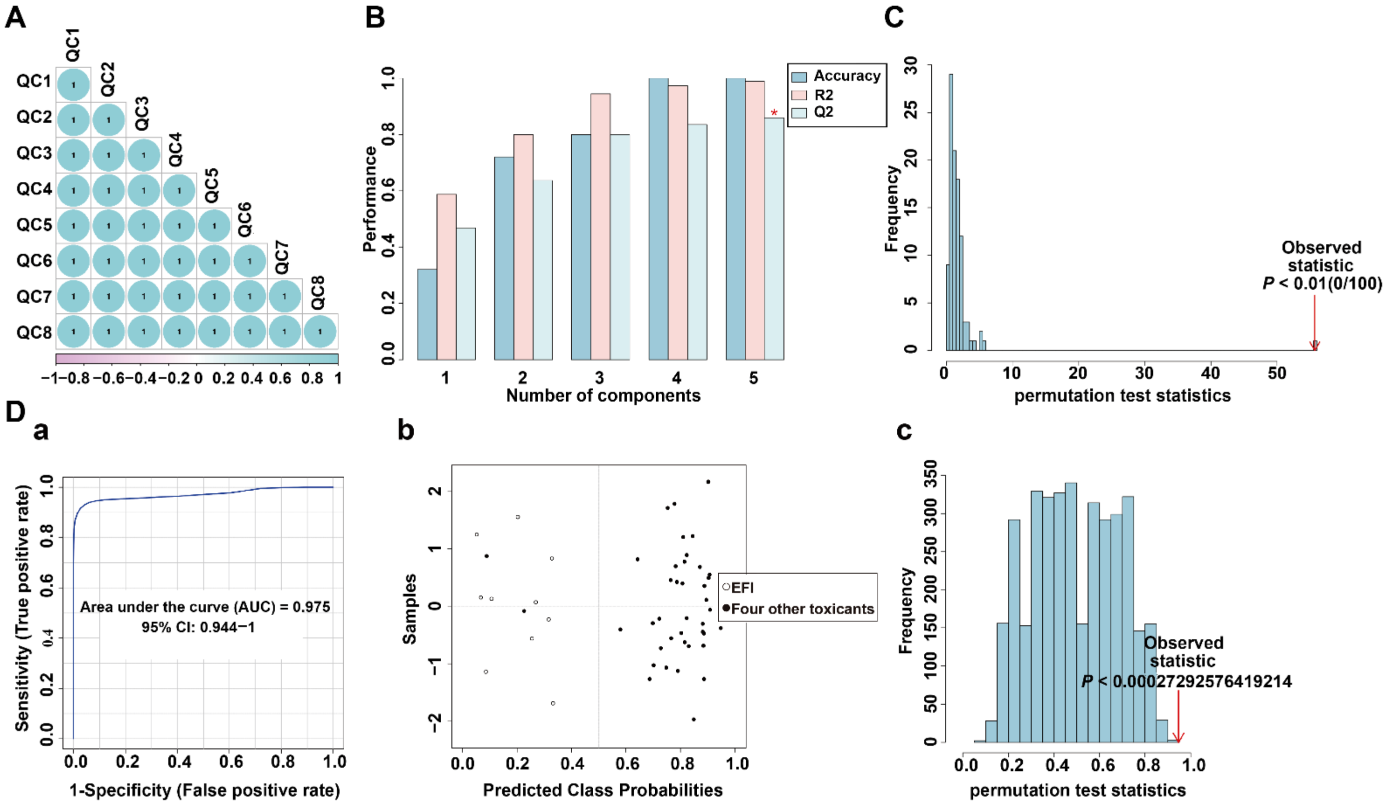

Figure S2. Evaluation of PCA and PLS-DA models for brainstem samples and the results of EFI classification model validation. A: Correlation analysis plot of QC samples. B: PLS-DA model

cross-validation plot by 10-fold CV algorithm, the five-component model was the best (marked ∗), R2 = 0.98959 and a Q2 = 0.85946. C: PLS-DA model with 100 permutation-test plots, $p < 0.01$. D: **B**: Predictive performance of classification models in the EFI group relative to the four other toxicants fatal intoxication models (Heroin, DL-METH, CO, CO2, $n_{animal} = 10$ respectively) in brainstem samples. (**a**) The ROC plot shows that the AUROC value is 0.975 (95% CI: 0.944−1) (**b**) The confusion matrix shows that two cases in the control group were wrongly diagnosed as EFI. (**c**) Classification model with 100 times permutation test plot, $p < 0.01$.

To further verify the specificity of this classification model in brainstem samples, four more toxicant lethal models (Heroin, DL-METH, CO, CO2) were also introduced for the above ROC testing. The results of the ROC plot (AUC = 0.975) and the distribution states of the confusion matrix plot showed that the classification model could well distinguish the EFI group from the other four toxic poisoning lethal groups, and the result of the permutation test ($P < 0.01$) shows that the model was not overfitted (Figures S2Da–c). The classification model exhibits high specificity in brainstem samples.

Table S8. AUC values of different candidate differential metabolite combinations in brainstem tissue samples.

**Candidate differential metabolites**	**Combinations of different candidate differential metabolites**			
**Combined Log FC > 1, or < -1, *P* < 0.05**	**1**	**2**	**3**	**4**
Palmitic acid	√		√	√
Indole-3-lactic acid	√	√		√
Creatine	√	√	√	
Indole-3-acrylic acid		√	√	√
AUC-value	0.999	0.994	0.998	0.991

Table S9. Prediction results of new brainstem samples (Validation set).

**Sample No.**	**Probability**	**Class**
**1**	0.98832	EFI
**2**	0.97902	EFI
**3**	0.99817	Control
**4**	0.99585	Control
**5**	0.95477	Control
**6**	0.93889	Control
**7**	0.99797	Control
**8**	0.99834	Control
**9**	0.91156	Control
**10**	0.90506	Control

Table S10. Sensitivity validation results of classification models in estazolam different dose groups (brainstem samples).

	**6 x LD_{50}**	**3 x LD_{50}**	**100 x**	**50 x**
**AUC-value**	0.999	1	0.908	0.872
TP	10	10	8	8
TN	39	40	33	34
FP	1	0	7	6
FN	0	0	2	2
**Precision (PPV)**	0.9756	1	0.8372	0.8421
**NPV**	1	1	0.8919	0.8095
**Recall**	1	1	0.8000	0.8000
**F1-score**	0.9877	1	0.8182	0.8205

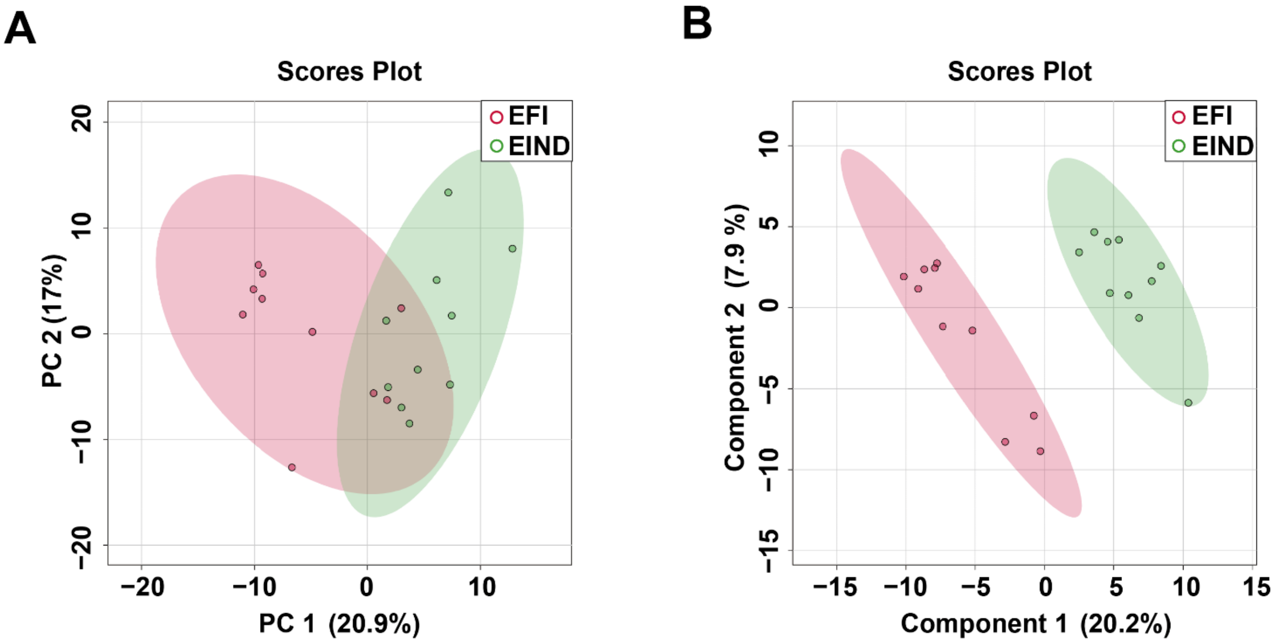

Figure S3. Overview of metabolic profiles of the EFI and EIND (n_{animal} = 10, respectively) groups. A: PCA score plot. B: PLS-DA score plot.

Table S11. Endogenous differential metabolites in the EFI group vs the EIND group.

FC > 1.5 or FC < 0.67, *P* < 0.05	FC	*P*	VIP	Trend
N-Arachidonylglycine	2.6661	0.00010073	1.79766	up
NADH	2.6048	0.00034098	1.70344	up
Adenosine diphosphate ribose	2.3382	0.0013691	1.57474	up
Stearoyl ethanolamide	2.0853	0.037478	1.20522	up
N-Acetylvanilalanine	1.8335	0.0006948	1.64066	up
Acetylcarnitine	1.7892	0.0077646	1.3639	up
(R)-3-hydroxybutyrylcarnitine	1.591	0.00045466	1.71114	up
Adenine	1.5507	0.0017718	1.56552	up
Uric Acid	1.5027	0.00050132	1.6744	up
15,16-DiHODE	0.66153	0.016455	1.30794	down
3-Hydroxydecanoic acid	0.61526	0.0021894	1.53144	down
D-lysopine	0.60846	0.00023753	1.73162	down
Kynurenine	0.58367	0.038273	1.11644	down
2-Hydroxycaproic acid	0.56784	0.0076504	1.385	down
D-Gluconic acid	0.53105	0.00057824	1.65746	down
3-Oxotetradecanoic acid	0.51422	4.4×10^{-5}	1.8536	down
alpha-Aminoadipic acid	0.49592	8.47×10^{-6}	1.99162	down
Indole-3-lactic acid	0.49278	0.0050607	1.44342	down
Leu-Val	0.47859	0.00040455	1.7118	down
3-Hydroxysebacic acid	0.45214	0.0022586	1.5244	down
Arg-pro	0.43829	0.0004477	1.6837	down
Isovalerylglutamic acid	0.42847	0.0097986	1.34168	down
Homovanillic acid sulfate	0.286	0.0031413	1.4989	down
Hippuric acid	0.28531	0.00048126	1.6788	down

Table S12. Results from Quantitative Enrichment Analysis of EFI group vs EIND group.

Metabolite Set	Total	Hits	Statistic	Expected	*P*	Holm *P*	FDR
Lysine degradation	25	2	67.684	5.2632	8.47×10^{-6}	5.08E-05	5.08×10^{-5}
Purine metabolism	65	3	45.623	5.2632	3.12×10^{-5}	1.56E-04	9.35×10^{-5}
Phenylalanine metabolism	10	1	50.091	5.2632	4.81×10^{-4}	0.001925	8.67×10^{-4}
Pentose phosphate pathway	22	1	49.109	5.2632	5.78×10^{-4}	0.001925	8.67×10^{-4}
Pyrimidine metabolism	39	1	24.023	5.2632	0.028244	0.056487	0.033892
Tryptophan metabolism	41	1	21.735	5.2632	0.038273	0.056487	0.038273

Table S13. Quantification of metabolites in brainstem tissue samples(ng/mg).

	Control	EIND	EFI
**Lysine**	0.528432	0.717235	1.092008
	0.625512	0.720427	1.340978
	0.583638	0.755141	0.937166
	0.687387	0.746447	1.166628
	0.524275	0.797177	0.841333
	0.4395	0.740366	1.007781
	0.570175	0.837613	1.153898
	0.700907	0.660676	0.909991
	0.698202	0.685484	1.14234
	0.555585	0.754073	1.298486
**Saccharopine**	0.755505	1.010357	1.107189
	0.750406	0.742726	1.085818
	0.801162	0.881176	1.089089
	0.44937	0.734578	1.518584
	0.765673	1.030368	1.073638
	0.617363	0.898919	1.108023
	0.616964	1.458254	1.443831
	0.671605	1.09252	1.243551
	0.488346	0.800901	1.048675
	0.761186	0.883453	1.807271
